# Measuring Changes in Consumer Resource Availability to Riverine Pulsing in Breton Sound, Louisiana, USA

**DOI:** 10.1371/journal.pone.0037536

**Published:** 2012-05-30

**Authors:** Bryan P. Piazza, Megan K. La Peyre

**Affiliations:** 1 School of Renewable Natural Resources, Louisiana State University Agricultural Center, Baton Rouge, Louisiana, United States of America; 2 U.S. Geological Survey, Louisiana Fish and Wildlife Cooperative Research Unit, School of Renewable Natural Resources, Louisiana State University Agricultural Center, Baton Rouge, Louisiana, United States of America; University of Otago, New Zealand

## Abstract

Resource pulses are thought to structure communities and food webs through the assembly of consumers. Aggregated consumers represent a high quality resource subsidy that becomes available for trophic transfer during and after the pulse. In estuarine systems, riverine flood pulses deliver large quantities of basal resources and make high quality habitat available for exploitation by consumers. These consumers represent a change in resources that may be available for trophic transfer. We quantified this increased consumer resource availability (nekton density, biomass, energy density) provided by riverine flood pulsing in Breton Sound, Louisiana, USA. We used water level differences between an area subject to two experimental riverine flood pulses (inflow) and a reference area not receiving inflow to identify the percentage of nekton standing stock and energy density that may be attributable solely to riverine pulsing and may represent a consumer resource subsidy. Riverine pulsing accounted for more than 60% of resident nekton density (ind m^−2^), biomass (g m^−2^), and energy density (cal m^−2^) on the flooded marsh surface during two experimental pulse events in 2005. Our results document the potential subsidy of resident nekton standing stock from a riverine flood pulse available for export to subtidal habitats. Given predicted large scale changes in river discharge globally, this approach could provide a useful tool for quantifying the effects of changes in riverine discharge on consumer resource availability.

## Introduction

Resource pulses affect many terrestrial and aquatic systems and are thought to structure communities and food webs through the assembly of consumers, particularly generalist species [1]–[5]. Aggregated consumers represent a resource subsidy that becomes available for trophic transfer during and after the pulse. Several examples provide evidence of allochthonous resource subsidies propagating through secondary consumers in terrestrial and aquatic systems [6]–[11], yet there are few studies that document the value of the change in consumer resource availability that is made available by an estuarine resource pulse [12].

Estuaries are pulsing ecosystems that receive periodic cross-border subsidies of both marine and terrestrial origin [13]–[14]. One example of a marine resource pulse is that of migrating anadromous fish. Tremendous amounts of allochthonous energy are made available to the estuary annually by decomposing fish carcasses, and this nutrient energy can be tracked through the food web as consumers exploit the subsidy [15]–[17]. One of the most influential pulses affecting community structure and function in estuaries is freshwater inflow [13], [18]–[19]. Freshwater inflow delivers basal (terrestrial) resources to large expanses of estuarine habitat and also makes high-quality habitat available for exploitation by consumers that rapidly assemble to flooded habitats. These consumers represent a measurable resource subsidy that will become available to higher-order pelagic consumers after the pulse ends [13], [20]. In estuarine ecology, it is generally accepted that resident nekton species may be critical in this transfer and outwelling of energy across habitat boundaries and to near-shore systems through a process known as trophic relay (vascular plant material – microbes – invertebrates – fish, [10], [20]–[23]). Measuring the standing stock of these aggregated resident nekton during riverine flood pulses may provide a means to quantify the effects of the resource pulse and the resulting consumer subsidy available for trophic transfer to pelagic habitats.

Freshwater pulses can occur with large runoff events, but more often these pulses are the result of annual riverine flooding. Riverine flood pulses deliver large quantities of allochthonous resources to estuaries [24]. These resources are readily assimilated by secondary consumers [12], [25]–[28] and may be responsible for increased nekton growth, changes in community structure, and trophic diversity [28]–[31]. Riverine pulses are particularly important for recruitment of resident nekton consumers that key into these flood events for spawning and rapid growth [21], [29]–[30], [32].

Flood pulses in estuaries are highly variable, because they are dependent on factors that are often far removed from the estuary itself [33]. Flood pulses are driven both by variability in climate (e.g., ENSO, climate change) and land use (e.g., dams, water withdrawals) that affect flow [19], [31], [34]–[36], and future forecasts of climate warming and increased development predict large scale changes in river discharge on every continent [36]. This additional variability in river flow will necessitate more management interventions and restoration to protect ecosystems [31], [36]. Consequently, there is a demand for greater basic understanding of the value of riverine flood pulses for communities and food webs, because attempts to protect estuarine systems will have to include the maintenance and restoration of riverine flood pulses, including their inherent variability.

Direct measures of consumer aggregation (e.g., density, biomass, diversity, growth), and behavioral response (e.g., diet switching, trophic cascade effects) are commonly used to assess the effects of resource pulses on community and food-web dynamics [1], [4]–[6], [28]–[30], [32]. Although extremely valuable, these approaches fail to provide high-level production measures, such as energy density, that can be used for comparisons of habitat quality [37]. With this work, we propose to quantify the potential subsidy (nekton density, biomass, energy density) provided by riverine pulse events. We hypothesized that a riverine pulse event would increase consumer resource availability, providing a positive potential subsidy of nekton density, biomass and energy density. We use the term “potential” because we are measuring what might be available for actual transfer via predation, mortality, excretion (i.e., consumer resource availability) and, in this study, do not document actual transfer.

**Figure 1:**
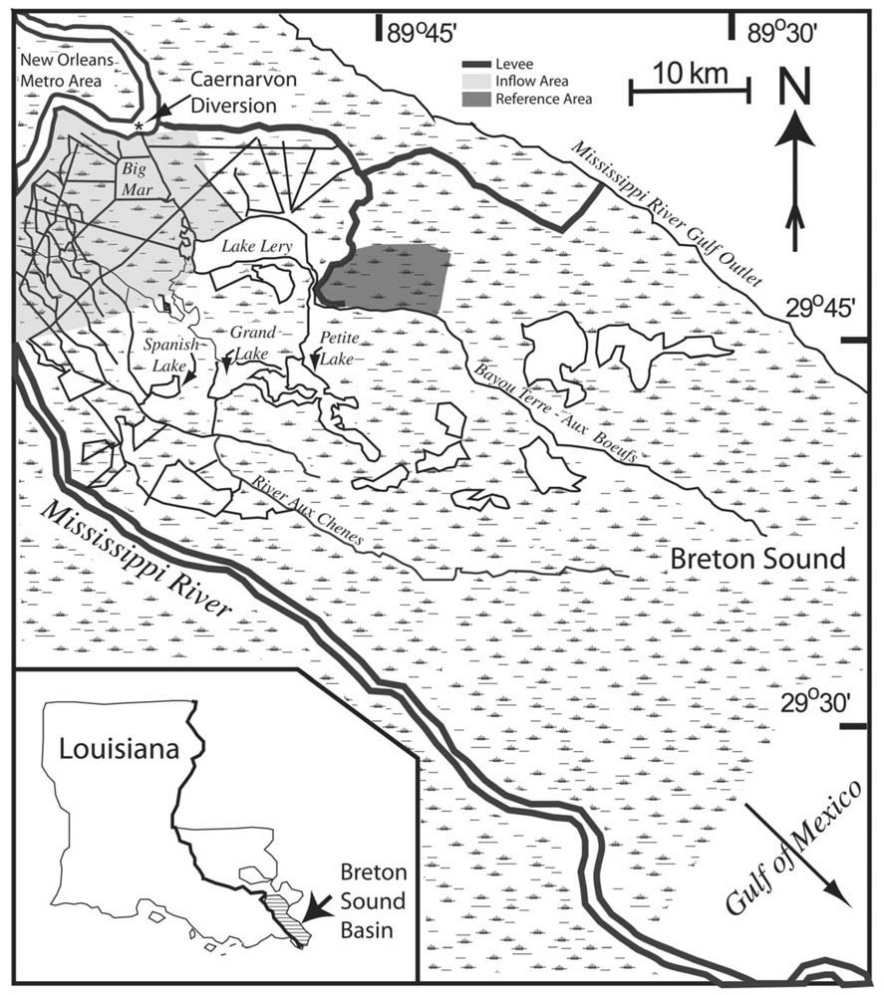
Breton Sound estuary, Louisiana, USA and the inflow (light gray) and reference (dark gray) areas used in this study. Also shown is the location of the Caernarvon Freshwater Diversion. Map adapted from [32], [39].

## Methods

### Ethics Statement

This work was conducted in accordance with institutional, national and international guidelines concerning the use of animals in research. The experimental protocol prepared for this study was approved by the Louisiana State University Institutional Animal Care and Use Committee – approval ID #05-008.

### Study Area

The study took place in upper Breton Sound estuary, Louisiana, USA, a 271,000 ha estuary in the Mississippi River deltaic plain of southeast Louisiana (Fig. 1). It is microtidal and consists of bays, lakes, bayous, canals, and fresh, intermediate, brackish, and saline marsh types, and a relict Mississippi River distributary (Bayou Terre aux Boeufs) that divides the basin geographically and hydrologically. Dominant emergent vegetation in upper Breton Sound consists of *Spartina patens* (Aiton) Muhl (Saltmeadow cordgrass) and *Schoenoplectus americanus* (Pers.) Volkart ex Schizz & R. Keller (Chairmaker’s bulrush).

The Caernarvon Freshwater Diversion structure is located at the head of the estuary and is capable of delivering substantial amounts of fresh water (227 m^3^ s^−1^), allochthonous sediments (1×10^8^ kg y^−1^) and nutrients to the basin [24], [38]. Caernarvon became operational in 1992 and was designed to moderate salinities and reintroduce controlled river inflows to Breton Sound. These controlled pulses release large fluxes of river water into the basin and are capable of inundating upper basin marshes west of Bayou Terre Aux Boeufs (∼ 5,700 ha; inflow area) for several days [32], [38]–[39]. Furthermore, upper basin marshes east of Bayou Terre Aux Boeufs are hydrologically separated from Caernarvon flow, and flooding there is dominated by meteorological forcing, providing a reference area subject only to flooding from meteorological forcing [26]. The ability to control inflow provided a unique opportunity to measure potential consumer resource subsidies, because it allowed for experimental flood pulses with control over the timing and duration of habitat flooding; the presence of adjacent habitat hydrologically separated from the inflow provided a reference area.

In this estuary, direct measures of consumer aggregation (e.g., density, biomass, diversity, growth), and behavioral response (e.g., diet switching, trophic cascade effects) have been shown to differ between the inflow and reference areas, and these effects have been attributed to riverine pulses through Caernarvon [25]–[26], [28]–[30], [32]. However, up to now, no quantitative estimates have been made of the potential energetic contribution of Caernarvon to secondary production in Breton Sound estuary.

**Figure 2:**
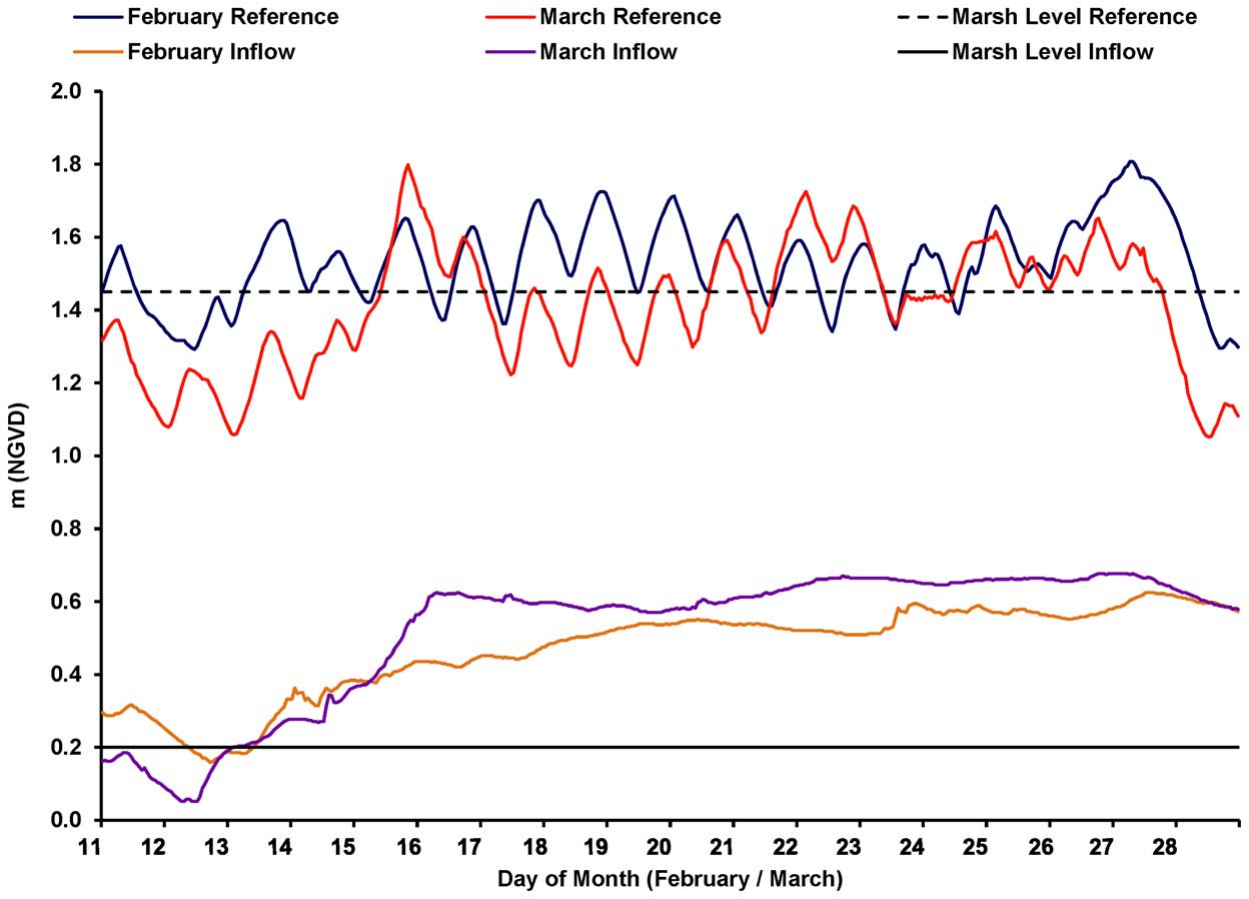
Water level and marsh elevations (National Geodetic Vertical Datum, NGVD) before and during two (February 14–28 and March 12–28) Caernarvon experimental high-pulse flow events into Breton Sound estuary in 2005. Because the reference area did not contain a surveyed water level recorder, stages and marsh level were based on an investigator-created datum, transferred from USGS 73745257 http://waterdata.usgs.gov/nwis/uv?format=gif&period=31&site_no=073745257; NGVD).

**Table 1:** Environmental characteristics in inflow marshes during two Caernarvon experimental high-flow pulses in February and March 2005.

Variable	February (n = 66)	March (n = 75)
Salinity (psu)	0.3±0.01 (0.2−0.7)	0.2±0.01 (0.2−0.2)
Dissolved Oxygen (ppm)	5.7±0.22 (1.8−9.4)	2.2±0.08 (1.0−4.3)
Water Temperature (°C)	17.3±0.35 (11.3−23.2)	18.4±0.50 (10.5−28.1)
Turbidity (NTU)	15.9±1.14 (0.8−50.0)	14.9±1.06 (0.9−38.3)

Data are expressed as mean ± SE (range).

### Data Collection

#### Nekton and environmental variables

Nekton samples and environmental variables were collected daily through two experimental riverine pulses in 2005 (February 14–28, March 12–28). Each day, sampling locations were selected randomly within the inflow area. Samples were collected with a 1.14-m cylindrical (1 m^2^) drop sampler in vegetated marsh habitat located in marshes downstream of the Caernarvon diversion structure. The drop sampler was suspended approximately 3 m from the bow of the boat and 1 m above the marsh surface by a telescoping aluminum boom. A drop sampler was chosen because it provides many advantages for sampling flooded marsh habitat including effectiveness at cutting through underground plant runners, high catch efficiency, and complete enclosure of the water column [40]. In addition, the telescoping boom allowed sampling in the flooded marsh 2–3 m from the edge where most nekton biomass on the marsh surface occurs [41]. Each sampling site was approached slowly and quietly. The outboard motor was shut off, and the boat was allowed to drift to the vegetated marsh edge. In areas that were too shallow to drift, investigators quietly pushed the boat into position. Once in position, the sampler was dropped and seated securely into the marsh substrate.

After the sampler was dropped, the location of the sampler was logged with a Garmin GPS III, and a suite of environmental variables was collected inside the sampler. Water temperature (°C), conductivity (ms), salinity (psu), dissolved oxygen (DO, mg l^−1^), and pH were measured with a handheld YSI 556 MPS (YSI Environmental, Inc.). Turbidity (NTU) was measured with a fluorometer (Aquaflour 8000, Turner Designs, Inc.). Five water depth measurements (mm) were taken inside the sampler with a meter stick, and hourly water depth was downloaded from nearby recorders (Inflow area – USGS 073745253, http://waterdata.usgs.gov/usa/nwis/uv?site_no=073745253; Reference area – USGS 073745257, http://waterdata.usgs.gov/nwis/uv?format=gif&period=31&site_no=073745257). Percent vegetative composition of emergent vegetation was visually estimated inside the sampler, and stems were clipped at the substrate and returned to the lab where they were identified and sorted by species and counted.

After environmental variables were measured, nekton inside the drop sampler were collected with 10 successive dip net sweeps (in opposite directions) by two investigators concurrently. After dip netting, retained water was pumped through a 1-mm mesh plankton net into a 1-mm mesh cod-end bag (Sea-Gear Corp., Melbourne, FL). Remaining organisms were removed from the marsh substrate by hand. Organisms were preserved in 10% formalin and returned to the laboratory for processing.

In the laboratory, samples were sorted, and nekton were identified to the lowest feasible taxon and counted. Total length of fish, shrimp, and crayfish and carapace width of crabs were measured to the nearest mm. Individuals of each species in a sample were pooled and weighed (g wet weight) to determine biomass.

**Table 2:** Wet weight biomass (WW; g) to dry weight biomass (DW; g) relationships and mean energy density (cal g^−1^ DW) for six dominant resident nekton species caught weekly on the flooded marsh surface in upper Breton Sound estuary, Louisiana throughout an experimental riverine flood pulse in spring 2007.

		Wet Weight - Dry Weight				Energy Density	
Species	Common Name	n	Equation	r^2^	Mean (SE)Water Content	n (pellets)	Mean (SE)cal g^−1^ DW
*Palaemonetes paludosus*	Riverine grass shrimp	392	DW = 0.190571(WW)	0.81	0.81 (0.002)	181 (8)	6141.85 (45.01)
*Heterandria formosa*	Least killifish	167	DW = 0.205544(WW)	0.66	0.81 (0.008)	146 (2)	6626.12 (7.48)
*Gambusia affinis*	Mosquitofish	2193	DW = −0.002934+0.206633(WW)	0.91	0.81 (0.001)	921 (36)	6294.70 (48.42)
*Lucania parva*	Rainwater killifish	80	DW = −0.01548+0.241053(WW)	0.90	0.81 (0.003)	60 (4)	5790.55 (204.37)
*Poecilia latipinna*	Sailfin molly	164	DW = −0.005112+0.230281(WW)	0.98	0.78 (0.002)	46 (5)	6435.60 (49.51)
*Cyprinodon variegatus*	Sheepshead minnow	39	DW = −0.014876+0.215689(WW)	0.99	0.81 (0.003)	24 (3)	5421.80 (9.99)

All relationships are statistically significant at p<0.0001. Equations with no intercept indicate that the intercept was not significantly different from zero.

#### Nekton energy density and standing stock

Nekton energy density of the six dominant species captured in 2005 sampling was determined using samples of these species collected during a 2007 experimental flood pulse specifically for this analysis. Because we only had wet weights from the 2005 sample data, we needed to determine species specific energy density (cal g^−1^), and species specific wet:dry weight equations. Using dip nets, individuals were collected weekly through an extended experimental flood pulse in 2007 (January 26– March 12). Immediately upon capture, nekton were frozen in an ice slurry and transported back to the laboratory. Each individual was then measured (total length, mm), weighed (wet weight, g), dried to a constant weight (50°C, 48 h), and re-weighed (dry weight, DW g). For each species, wet:dry weight equations were determined with simple linear regression (Model dry weight = wet weight). Conspecifics from each sample were pooled and pulverized. Analysis of energy density was done on one gram DW nekton pellets (Parr model 2811 pellet press) with a Parr 6200 isoperibol oxygen bomb calorimeter. Each pellet represented numerous individuals (>3) of a particular species collected in a specific sample, and the number of fish used for each pellet was dependent on the size of the individuals caught. Three replicate pellets were analyzed for each species and sample (i.e., date) combination if enough tissue powder (3 g) was available. In the event that enough powder was not available, we analyzed as many pellets as possible.

The standing stock nekton energy density was estimated using the calculated energy density (above) of the six dominant resident nekton species that assembled to flooded marsh habitat during 2005 flood pulse events (*Palaemonetes paludosus*, *Heterandria formosa*, *Gambusia affinis*, *Lucania parva*, *Poecilia latipinna*, *Cyprinodon variegatus*, [32]). Dry weight biomass (g m^−2^) of each species in each 2005 sample was multiplied by its respective energy density (cal g^−1^), as calculated below, and then the product was summed to derive specific energy density estimates (cal m^−2^) for each 2005 sample. This number represented the standing stock nekton energy per square meter of flooded marsh surface in the inflow area during 2005 riverine flood pulses.

#### Nekton subsidy calculations

We defined the potential nekton standing stock and energy density subsidies as the number (ind m^−2^), biomass (g m^−2^), and energy density (cal m^−2^) of nekton present on the flooded marsh surface in the inflow area that exceeded what would have been found without the freshwater pulse event. To determine actual flooding that we could attribute to the freshwater pulse, we used water levels in the reference area as a guide to inform what water levels would have been in the inflow area without the diversion, and this guide was used to develop nekton subsidy calculations (described below). Specifically, we used water level differences between the inflow and reference areas to identity the percentage of nekton standing stock and energy density in inflow marshes that may represent a resource subsidy provided by two experimental Caernarvon river pulses.

To quantify these subsidies, it was first necessary to separate samples taken in the inflow area into subsidized and unsubsidized categories. Subsidized samples were defined as those that met one of the following two conditions. They were taken in the inflow area either (1) when the reference area was not flooded, or (2) when the reference area was flooded, but the mean water level in the inflow area was greater than the maximum mean depth of flooding in the reference area. This approach assumed that without the freshwater flow from Caernarvon, the upper inflow basin would have flooded simultaneously to and with approximately the same characteristics as the reference basin.

Next, we calculated estimates of the potential nekton subsidy for each pulse event (February/March) by determining the proportion of total nekton density, biomass, and nekton energy accounted for by subsidized samples (i.e., X(subsidized samples)/X(total samples), where X = nekton density, biomass or energy density). Finally, these proportions were averaged across pulses and then multiplied by the mean density, biomass and energy estimates from the inflow area to calculate estimates of nekton standing stock (ind m^−2^, g m^−2^) and potential energy subsidy (cal m^−2^) that could be considered to be due to the riverine inflow.

**Table 3:** Mean (±SE) standing stock nekton density (ind m^−2^) and nekton energy density (cal m^−2^) for the six dominant nekton species that assembled to the flooded marsh surface in the inflow area during two (February and March 2005) experimental riverine flood pulses in upper Breton Sound estuary, Louisiana.

		February (n = 66)		March (n = 75)	
Species	Common Name	Density	Energy Density	Density	Energy Density
*Palaemonetes paludosus*	Riverine grass shrimp	28.8±5.3	4215.7±765.8	15.2±4.2	3511.4±936.6
*Heterandria formosa*	Least killifish	12.4±2.4	590.4±106.8	32.6±11.8	2312.8±1058.3
*Gambusia affinis*	Mosquitofish	11.2±3.6	700.7±226.8	10.5±2.2	1511.6±368.0
*Lucania parva*	Rainwater killifish	8.5±2.5	1427.5±397.2	3.2±0.5	537.8±146.2
*Poecilia latipinna*	Sailfin molly	2.9±0.7	293.1±110.9	2.6±0.5	553.1±168.1
*Cyprinodon variegatus*	Sheepshead minnow	2.9±0.8	227.3±126.1	2.9±0.5	446.6±129.8

## Results

### Nekton and Environmental Variables

A total of 141 samples were collected within the inflow area during the pulse events. During sampling, salinity, temperature, dissolved oxygen, and turbidity were within the normal range of estuarine variability in this region (Table 1). Furthermore, during sampling, the marsh was flooded 69% (February pulse) and 64% (March pulse) of the time in the inflow area, and only 31% (February pulse) and 14% (March pulse) of the time in the reference area. Subsidized samples comprised 91% (n = 60) and 92% (n = 69) of the inflow samples collected in February and March, respectively. Flooding in the reference area was driven solely by meteorological and tidal forcing during the time of sampling; in contrast, the freshwater inflow obscured tidal periodicity in the inflow area soon after diversion pulsing began (Fig. 2).

A total of 5,948 individuals of 16 taxa were collected in the inflow area during sampling. Six species – *Palaemonetes paludosus* (n = 2,363), *Heterandria formosa* (n = 1,975), *Gambusia affinis* (n = 743), *Lucania parva* (n = 377), *Poecilia latipinna* (n = 81), *Cyprinodon variegatus* (n = 70) – comprised 95% of the total abundance and were selected for use in calculations of nekton energy density. Information on the total nekton community sampled can be found in [32].

### Nekton Energy Density and Standing Stock

A minimum of 24 individuals of each of the six dominant species were caught in 2007 and used to determine energy density and wet;dry weight regressions (Table 2). Regression analysis revealed highly significant dry weight conversion equations for the six dominant resident fish species, and mean water content of all species was similar (78–81%). Energy density values for resident nekton ranged from 5,410–6,718 cal g^−1^, with highest mean energy density reported for *Heterandria formosa* (Table 2).

Standing stock density of the six dominant resident nekton species in inflow marshes ranged from 2.6–32.6 ind m^−2^ during the freshwater pulses (Table 3). These assembled individuals represented an available stock of transferrable energy on the flooded marsh surface that ranged from 227.3–4215.7 cal m^−2^. During both pulses, standing stock of *Palaemonetes paludosus* was greatest. There was also intraspecific variability in the available standing stock between pulses. For example, standing stock density of *Heterandria formosa* increased almost threefold, and its energy density increased almost fourfold from February to March pulses.

### Potential Nekton Energetic Subsidy

Subsidized samples represented 67.6±17.1% of the standing stock nekton density and 61.1±12.1% of the nekton biomass in the inflow marshes during the 2005 riverine flood pulses (Table 4). Additionally, inflow marshes contained over 8,000 cal m^−2^ of standing stock resident nekton energy, of which 61.7±15.8% was subsidized by the flood pulses.

**Table 4:** Mean (SE) standing stock of resident nekton – density (ind m^−2^), biomass (g DW m^−2^), and energy density (cal m^−2^) – that assembled to the flooded marsh surface in the inflow area during two (February and March 2005) experimental riverine flood pulses in upper Breton Sound estuary, Louisiana.

		Nekton Subsidy	
	Mean (SE) in Inflow area	%	Mean (SE) attributed to resource pulse
Resident Nekton Density (ind m^−2^)	40.0 (7.2)	67.6	27.0 (4.8)
Nekton Biomass (g DW m^−2^)	1.3 (0.2)^1^	61.1	0.8 (0.1)
Nekton Energy Density (cal m^−2^)	8164.0 (1490.0)	61.7	4990.8 (895.6)

Also shown is the estimated nekton standing stock that was a subsidy attributed to the experimental pulses. Mean standing stock is based on nekton community data reported in a previous study [32]. Dry weight biomass was calculated with equations in Table 2. Errors for potential nekton subsidy were calculated using the formula (Z_err_ = Z[(X_err_/X)^2^(Y_err_/Y)^2^]^1/2^.

## Discussion

Managed riverine pulsing at the Caernarvon freshwater diversion structure increased potential consumer resource availability by as much as 60% as measured by increased nekton density, biomass, and energy density. These marshes receiving freshwater pulses contained over 8,000 cal m^−2^ of standing-stock resident nekton energy that was available for export and assimilation by higher trophic levels as water levels receded. This increase in consumer resource availability quantifies potential subsidies that could contribute to system energetics via transfer through numerous mechanisms including predation, mortality, or excretion [3], [5]–[6], [9].

While our approach was able to document an increase in consumer resource availability, it did not identify the specific underlying mechanism(s) (i.e., increased marsh access, increased food supply) responsible for this. There is debate among estuarine ecologists whether the positive effects of freshwater flow on consumer populations are linked to bottom-up controls (e.g., Apalachicola Bay [42]), simply the change in physical habitat attributes that occur with flooding (e.g., San Francisco Bay [43]), or both. In this study, we were able to demonstrate clearly that these Caernarvon pulses provide potential trophic subsidies, as measured by nekton density, biomass and energy density that can be attributed to flooding from the pulse event suggesting that physical habitat changes may be one of the key mechanisms. This finding is supported by past research in this system demonstrating that increased nekton density and community changes are associated with increased water levels during pulse events [30], [32] or changes in salinity [28], [30]. At the same time, other studies in this system demonstrate that these riverine flood pulses deliver a significant amount of basal resources (140 µM of total N, 5 µM total P) at a level that is directly comparable to other allochthonous resource pulses in aquatic habitats (migratory salmon, [8]; migratory waterfowl, [44]; cicada carcasses, [9]) and that these nutrients are rapidly assimilated in the upper estuary [24]. Studies in this estuary also show that riverine nutrients propagate into the resident nekton consumers [25]–[26], [28]–[29] supporting the bottom-up control concept. However, we did not quantify the actual transfer of this subsidy to the higher-order consumers (i.e., predation). Combined, this evidence suggests that both bottom-up control and change in physical habitat attributes may play a role in impacting consumer resource availability; however, we still lack specific evidence of actual trophic transfer [25]–[26], [28]–[30], [32].

Several different models have been proposed to explain how resource pulses structure communities and food webs (i.e. trophic relay, onion model, spider web model, internet model, constant connectance hypothesis; [21], [45]–[46]). In this estuary, this study and others suggest that a relatively small number of resident species contribute disproportionately to changes in the food web, which conceptually supports the simplified food web models (i.e., trophic relay; [25]–[26], [28]–[30], [32]). While this study showed that relatively few resident species formed the core of the potential energy available for trophic relay, we did not investigate actual food web transfer. However, during this study we routinely observed red drum (*Sciaenops ocellatus*), a species predominantly found in brackish to marine waters, cruising alongside the flooded marsh edge, outside their preferred salinity regime (salinity <1.0; [32]). These observations suggest that aggregated nekton on the flooded marsh surface provided a high-enough quality food source to lure them into a suboptimal environment. These observations would appear to concur with other studies suggesting that under seasonal (predictable) flood pulse regimes, some species will tolerate sub-optimal conditions to exploit predictable resources [47]–[48]. Future research on the effects of resource pulses should be directed toward unraveling specific food-web interactions with a combination of field sampling and experimental manipulation in order to determine such things as the response of highly mobile pelagic and avian predators [2], [9], [23], energy flow and interaction between nekton consumers and invertebrate prey [22], lags in assembly and nutrient transfer [9], and community response [2].

It has been suggested that aggregation represents only half of the consumer response to resource pulses, and that reproduction may have more persistent effects on local communities [2], [5]. Resident nekton species, particularly poeciliids, have been shown to be intimately tied to seasonally flooded wetland habitats in both forested wetlands and coastal marshes, as spawning, and breeding episodes typically coincide with flood events [21], [49]. We did not document reproductive status of resident nekton during riverine pulse events; however, our energy density results suggest that aggregated nekton may have been reproducing in flooded marshes. Reproductive status has a disproportionate effect on caloric value because the gonadal tissue is comprised largely of lipids and contributes to high overall energy values during reproduction [50]–[52]. Seasonal variability in energy density corresponding to reproduction has been shown for clupeids [15], [51], [53], salmonids [52], and gadids [54]. In poeciliids, it has been shown that as *Gambusia affinis* females increase in size, energy density increases, and the majority of the total energy is contained in ovaries and developing embryos [50]. High energy values for the nekton species in our study, particularly for *Heterandria formosa*, may reflect the onset of breeding, as reproduction in estuarine residents is particularly keyed to flooding events [21]. Future work should involve an assessment of the reproductive status of individuals during sampling.

While, clearly, actual nekton subsidy likely is affected by characteristics and timing of the riverine pulsing, the density and biomass values calculated for this study are directly comparable to those reported in other wetland systems [26], [55]–[56]; furthermore, when expressed as percentages, the subsides calculated in this study are similar to cross-border subsidies calculated in other systems (e.g., terrestrial insect subsidies comprise 27–60% of prey volume of some fish species [6]; terrestrial invertebrate prey subsidies comprise ∼ 50–70% of salmonid diets [11], [57]). These subsidy numbers were calculated for experimental spring pulses that were designed to mimic historic seasonal flood pulses, and thus possibly captured subsidies that reflect evolved responses [48]. In contrast, aseasonal flood pulse regimes may not elicit similar numbers or may require examination of different species. Maintenance of natural flood patterns thus may be critical in sustaining these subsidies and timing of managed flood pulses may have important food web implications.

Energy density estimates may provide a meaningful and comparable assessment of the energetic value of higher-order resource subsidies. Although estimates of energy flow may be inappropriate for understanding the effects of resource pulses on community dynamics [58], gross energy content is not only a useful measure of physiological status, health, and changes in habitat quality but is also a high-level production-related measure of the direct link between coastal marsh habitat and fishery production [37], [59]–[61]. Therefore, the potential energy density subsidy may be a good measure of the effect of resource pulses on habitat quality and a comparable measure of the change in consumer resource availability that results from resource pulses.
